# News and Notes

**Published:** 1908-05

**Authors:** 


### NEWS AND NOTES

An international committee has been appointed to take charge of the awarding of the medal to be awarded periodically as a memorial to Schaudinn, to the individual who has contributed most to the progress of microbiology.

On May 19th the alumni of the medical department of Tulane University propose giving a jubilee to celebrate the anniversary of the fiftieth year of teaching service of Prof. Chaille. On this occasion it is proposed to announce the establishment of a Chaille Memorial Fund, created to memorialize the occasion of Dr. Chaille's retirement from the medical department and to perpetuate his name.

---

"Dr. A. N. Richards of the College of Physicians and Surgeons of New York City has been appointed professor of Pharmacology in Northwestern University Medical School."

---

Dr. John B. Murphy has resigned as Professor of Surgery and co-head of the Department in Rush Medical College and has accepted the Professorship of surgery and head of the department in Northwestern University Medical School and position of attending surgeon at Mercy Hospital."

---

"Dr. A. W. Meyer of the University of Minnesota and formerly of Johns Hopkins has acepted the professorship of Anatomy in Northwestern University Medical School."

---

At the thirty-fifth annual meeting of the Florida Medical Association, held in Ocala, April 15-17, the following officers were elected: Dr. James F. McKinstrey, Jr., Gainesville, president; Drs. James, Dr. Love, Jacksonville, and William H. Powers, Ocala, vice-presidents; Dr. J. D. Fernandez, Jacksonville, secretary-treasurer (re-elected); Dr. Charles E. Terry, Jacksonville, librarian (re-elected), and Dr. J. Harris Pierpont, Pensacola, delegate to the American Medical Association.

---

The Kentucky State Association of Railway Surgeons will hold the 4th Annual meeting in Louisville, Ky., on May 12th and 13th, 1908.

The L. and N. R. R., and other lines reaching Louisville, will give passes upon request of their respective local surgeons.

### MISSISSIPPI VALLEY MEDICAL ASSOCIATION.

The thirty-fourth annual meeting of the Mississippi Valley Medical Association will be held in Louisville, Ky., October 13, 14, 15, 1908, under the presidency of Dr. Arthur R. Elliott, of Chicago.

Announcement has just been made of the selection of the orators for the coming meeting, by the President. The Address in Medicine will be delivered by Dr. George Dock, Professor of Medicine in the University of Michigan, Ann Arbor; and the Address in Surgery by Dr. Arthur Dean Bevan, Professor of Surgery in Rush Medical College, Chicago. The mere mention of these names is enough of a warrant that this feature of the program will be in every way first-class.

The local Committee of Arrangements in Louisville has selected The Seelbach hotel as headquarters, the general sessions and the section meetings being held in the hotel's large auditoriums.

One of the features of the entertainment projected is a smoker in the famous Rathskeller of the hotel—the finest of its kind.

The McDowell button, so much admired at the 1897 meeting in Louisville, will be reproduced in bronce for this meeting.

---

### THE MEDICAL ASSOCIATION OF GEORGIA.

**The Medical Association of Georgia held its Fifty-Ninth** Annual Session at Fitzgerald, April 15, 16 and 17. Although the place of meeting was not in a central portion of the State, in spite of the inconvenient railroad schedule from many points, the attendance was very good, and the quality of the papers was excellent. The assembly hall was also well attended during the reading of all the papers—there being no social diversions to distract attention from the scientific part of the program.

Much credit is due Dr. M. A. Clark for the business-like manner in which the affairs of the Association were transacted.

### WEDNESDAY, APRIL 15.

Morning Session, 9:30 O'clock.

Prayer—By the Rev. J. C. Flanders.

Address of Welcome in Behalf of the City—Mayor J. G. Knapp.

Address of Welcome in Behalf of the Local Profession—L. S. Osborne.

Response to Address of Welcome—R. B. Barron, Macon.

Report of the House of Delegates.

### READING OF PAPERS.

10:30 to 1 O'clock.

1. A Resume of 148 Cases of Typhoid Fever with Reference to the Efficacy of Therapeutic Fasting.

R. M. Harbin, Rome.

ABSTRACT.—The food question is the most important. Toxemia is more marked when the gastro-intestinal symptoms are present. The exceptional cases require liberal feeding. The typhoid bacillus is found to originate chiefly in the intestinal canal and lymphopoietic system. The battle ground of treatment lies in the intestinal canal, for the nourishment should not be allowed to increase saprophytosis. Toxin is more dangerous to cell life than inanition. Organs furnishing avenues of infection should be kept passive. Typhoid toxin does not necessarily produce high temperature, and excessive nitrogenous waste probably results from mixed infections. Toxemia is a greater cause of emaciation than lack of food. Food management will protect a patient from the usual dangers. As sthenic cases furnish the more violent types, fasting is more admissible. Intestinal complications are as common in ambulatory cases as in others. Hemorrhage furnishes a greater danger by setting up conditions favorable to sepsis. Symptoms are no guide as to the presence of intestinal lesions, and routine feeding is more or less necessary. Tendencies to relapse are more frequently shown in abortive cases. Tympanites causes an increased area for absorption, and a food surplus increases peristalsis. Scientific data prove that clinical diagnosis may be relied upon with a reasonable degree of accuracy. Fasting favors the course of a normal typhoid infection, which is usually mild, and should be applied in severe cases only. Fasting enhances the effect of hydrotherapy. Gelatin lessens nitrogenous waste, and prevents hemorrhage. There was a mortality of 4.7 per cent.

2. Chills in Typhoid Fever—C. W. Strickler, Atlanta.

3. Advantages of Wiring Femur Over Other Methods, with Report of Cases—Craig Barrow, Savannah.

4. A Case of Superfetation—R. V. Martin, Savannah.

5. The Diagnosis and Treatment of Bright's Disease—H. F. Harris, Atlanta.

6. The Necessity for the Proper Care of School Children's Eyes—Dunbar Roy, Atlanta.

7. The Surgery of the Tubes and Ovaries—Geo. A. Wilcox, Augusta.

8. Creeping Eruption—G. O. Brinkley, Savannah.

9. The Results of Vaccine Therapy in Acute and Chronic Infections—J. Edgar Paullin, Atlanta.

ABSTRACT.—Infections, acute and chronic, localized and general. Portal of entry. Methods employed by the body to resist the invasion of micro-organisms. Resistance. The presence in the body fluids of opsonin. The influence of opsonin on bacteria. Sensitized bacteria. Method of preparing vaccines and their standardisation. Trend of events happening on the injection of a vaccine. Relation of cases and results obtained by the use of vaccines. Conclusions.

10. A Plea for the More Frequent Use of Obstetric Forceps—J. H. McDuffie, Columbus.

ABSTRACT.—Special reference to pathological conditions that may be avoided by not allowing "nature to take its course" in many cases. More harm results to mother and infant from prolonged and needless delay than is ever traceable to the use of forceps in skillful hands.

11. Puerperal Eclampsia and Its Treatment—Jno. W. Daniel, Savannah.

12. Cholelithiasis—Willis Jones, Atlanta.

13. Early Operation as a Means of Preventing Complications and Reducing the Mortality in Appendicitis—J. L. Campbell, Atlanta.

ABSTRACT.—If all cases of appendicitis were operated upon during the first twenty-four or thirty-six hours the mortality would be almost nothing. Remarks on early papers and discussions before the Medical Association of Georgia. Statistics from Georgia hospitals and sanitariums. Report of some acute cases, and cases of abscess with drainage.

AFTERNOON SESSION.

2:30 to 5:30 O'clock.

14. Cause of Scrotal Hematocele.

ABSTRACT.—Etiology, symptoms, and diagnosis. Report of a case.

15. Report of a Case of Ingestion of Safety Pin by a Child Two Years of Age—Subsequent Passage by Bowel Without Symptoms—Whatley W. Battery, Jr., Augusta.

ABSTRACT.—Comments on quick passage of pin into bowel, as illustrated by skiagraph taken three hours after ingestion. Absence of pain in abdomen during passage. Treatment.

16. Hydrocele and Spermatocele, with Report of Cases—W. L. Champion, Atlanta.

ABSTRACT.—Varieties, cause, diagnosis, frequently confused with other diseased conditions; the frequency of syphilitic involvement of the testicle as a cause of hydrocele. Carbolic injections, and when they should be used.

17. Early Operation for Adenoids—A. W. Stirling, Atlanta.

ABSTRACT.—Besides the "stomach attacks," disturbed sleep, aphosexia, earache, perforation of the tympanic membrane, deafness, mastoiditis and diminished power of resistance which may follow upon adenoids, a continuation of mouth breathing from nasal obstruction is likely in early life, and especially at the time of the second dentition, to produce permanent deformities. The palate is narrowed and elevated through compression of the upper jaw unopposed by the tongue, which plays an important part in normal conditions; the nasal septum is consequently bent; the nostrils are narrowed and immobile; the teeth are crowded; and pigeon-breast also results.

18. Colic, its significance—T. J. Charlton, Savannah.

ABSTRACT.—Colic is a disorder first met with in the newborn baby, occurs during all stages of life and is by no means uncommon in old age. Thus frequently met it becomes commonplace and like all things commonplace is more or less neglected. Colic on the other hand is often a marked and early symptom of serious and often fatal abdominal disease. The physician should constantly bear this in mind and before assuming that colic is merely the result of a minor disorder of digestion should first convince himself by exclusion that it is not the beginning of serious abdominal disease.

19. Respiratory Expansion—A Hitherto Neglected Factor in the Treatment of Pott's Disease—Michael Hoke, Atlanta.

ABSTRACT.—Attention is called to the necessity of permitting the chest to expand and develop while the patient is wearing a jacket for immobolizing the spine, and methods are shown how this may be done.

20. Mucus-Colitis and its Treatment—J. L. Farmer, Savannah.

21. Gonorrhœa—Thos. Chason, Donaldsonville.

22. A Favorable Report on the Use of Gonococcic Vaccine—E. G. Ballenger, Atlanta.

ABSTRACT.—During the past six months the author has been greatly impressed by the success he has been able to attain in the treatment of gonorrhœal conditions by injections of gonococcic "vaccine" or "bacterin," and embodies in this paper certain facts concerning this form of therapy and his impressions regarding its use. The clinical symptoms have been relied upon in selecting the time at which injections should be administered, and are believed to be of as much value in gonorrhœa as the "opsonic index." Of much importance is the treatment of all conditions and complications, so as to procure a free flow of fluids through the injected foci, so as to bring the juices laden with opsonins to the points where they are most needed.

23. The Practical Value of Cystoscopy, Ureteral Catheterization—Jabez Jones, Savannah.

24. Treatment of Epilepsy—Wesley Taylor, Atlanta.

ABSTRACT.—Care of general health necessary for the treatment of nearly all chronic diseases, and especially out of door exercise as shown in institutions where the patients are employed outside. The patients have fewer attacks when the weather permits them to do even hard manual labor and more attacks on Sunday than on week days. Principles on which bromide acts. Common salt, which is a nerve irritant, can be replaced in the human body by bromide, a nerve sedative, by proper dietary measures. Disadvantages to this method. Cases suitable to such treatment. Other methods of treatment. High blood pressure in epileptics. Advantages directed toward the lowering of blood pressure.

25. Neurasthenia—W. Herbert-Adams, Savannah.

ABSTRACT.—Its increasing prevalency, and the reasons therefor. Many of the assigned causes are in reality effects of the diseases. Necessity, for clearing differentinting neurasthenia from hysteria, hypochondria, melancholia and the organic neuroses. Therapeutics, include rest-cure, electricity, hydrotherapy, drugs, and, most of all, psychotherapy. Reasons why the neurasthenic falls an easy prey to Christian Science, osteopathy, advertising quacks, and other medical charlatans. Thoroughly imbue patient with your ability to cure him, then study each case carefully and treat it intelligently or let it alone, remembering that each failure makes the case far less amenable to future treatment.

26. Report of Five Cases of Facial Neuralgia Treated by Injections of Osmic Acid—Chas. C. Harrold, Macon.

ABSTRACT.—Four of above cases were typical, two were fresh cases and two were very severe casess of long standing. In all four of these the nerves were injected at their exits through the foramina on the face, the supra-orbital, infra-orbital and the mental. In no case were all three injected and in no case was it thought necessary to expose the nerves as recommended by Murphy. In all four of the typical cases the relief was immediate, absolute and lasting for varying lengths of time.

NIGHT SESSION—WEDNESDAY.

7:30 to 10 O'clock.

27. Weaning—H. McHatton, Macon.

ABSTRACT.—Breast milk is the best food for the infant. Modified cow's milk is the best obtainable artificial food. No proprietary food on the market can be given alone and continuously to an infant with safety. Weaning should always be gradual. The most important thing to observe with a baby is to keep an absolute record of its weight. Lactation and pregnancy are not incompatible.

28. Fermented Milk (Kefir): Its Origin and Therapeutic Application—L. Amster, Atlanta.

ABSTRACT.—Food among the Asiatic people, adopted by the medical profession in Europe—its value recognized in the

United States. Chemical analysis. Therapeutic application in acute diseases, fevers, diseases of the stomach and intestines, rheumatoid arthritis, Bright's disease, diabetes, pulmonary diseases, etc. Contra-indications.

29. Prealbuminuric Retinitis, with Report of Two Cases—H. H. Martin, Savannah.

ABSTRACT.—The relation between arterial fibrosis and atheroma. The relation between each of these and the various forms of nephritis, and whether or not there is another form of arterial degeneration as yet unclassified: namely, a hyaline degeneration.

30. Intestinal Toxemia—A. B. Simmons, Savannah.

31. Elimination and Alteration—B. P. Oliveros, Savannah.

ABSTRACT.—A reference to elimination in disease, especially in general septic conditions, toxemias, and the pregnant state by the use of any measures pertaining thereto, with a special reference to diaphoretics, diuretics and evacuants—also showing the close relationship of alteration, whereby certain drugs exert alterative action on the circulation, performing a dual office of assisting elimination, or making that process unnecessary.

32. Purpura Fulminans—R. P. Izlar, Waycross.

ABSTRACT.—Definition. Etiology and nature. Micro-organisms in the development of purpuric conditions, generally infectious conditions. Report of cases. Classification and course of the disease. Scope of drugs. Exaggerated mental conditions. Unfavorable prognosis.

33. The Use and Abuse of Irrigations—Wm. Perrin Nicolson, Atlanta.

34. Delayed Toxic Effect of Chloroform Anæsthesia—J. M. Sigman, Savannah.

35. A Case of Supra-Pubic Lithotomy—The Technique of Supra-Pubic Cystotomy and the After-Treatment—Eugene R. Corson, Savannah.

ABSTRACT.—The removal of eight stones weighing seven ounces; four of these stones forming a cup in which the other stones were free to move. The technique of supra-pubic cystotomy with special reference to the control of hemorrhage, the incision into the bladder, and the subsequent drainage and after-treatment generally. The use of a long catheter perforated in the middle which does the double duty of a catheter a temeure and supra-pubic drain. Paper illustrated by photograph of the stones and a figure showing the use of a combined catheter and supra-pubic drain.

36. Leucocytosis in Acute and Chronic Diseases—Walter S. Wilson, Savannah.

ABSTRACT.—Insufficiency in many cases of total count. The value of the differential count in diagnosis and prognosis to the surgeon and to the physician. Sources of error. Limitations. Interpretations must be made in the light of clinical conditions.

Exclusive Milk Diet for 12 Years—W. W. Terrell, Douglas.

THURSDAY, APRIL 16.

Morning Session, 9 to 1 O'clock.

President's Address.
Secretary's Report.
Report of House of Delegates.

READING OF PAPERS.

37. Long and His Discovery, with Comment on the Work Being done by the Federation of Women's Clubs for a Monument to His Memory—J. H. Goss, Athens.

ABSTRACT.—The above paper discusses the claim of Dr. Crawford W. Long, and recites the old story that he did the first operation in all the world under ether anesthesia. Makes a plea for the medical profession to join forces with the Federation of Women's Clubs and erect a monument to Long. This movement has already been inaugurated by the Athens Woman's Club.

38. Pneumonia—J. E. Sommerfield, Atlanta.

39. Lobar Pneumonia in Infants—Herman W. Hesse, Savannah.

40. The Indications for the Mastoid Operation—F. P. Calhoun, Atlanta.

ABSTRACT.—The relation that the mastoid bone bears to the intracranial structures is important for the general practitioner and surgeon to know, as well as the otologist. Permanent injuries to vital structures result from neglect. Usually the indications for the mastoid operations are evident, on account of pain, swelling, temperature, and aural discharge. Accurate history is first necessary. Inspection determining then if there is any swelling or redness over or about the mastoid. Furunculosis, or otitis externa, might cause this symptom. Swelling is the rule in children and infants, as it is an early sign; rare or a late symptom in adults. Palpation is the most valuable sign of mastoiditis, and it is the one sign for the general practitioner to make a diagnosis in the absence of other symptoms. The temperature is variable. Usually high in children; normal elevated one or two degrees in adults. The canal examination a method of examination more for the otologist than the practitioner; a bacteriological examination of the aural secretions is helpful. Blood examinations are of no practical value.

41. Antipyretics in Febrile Diseases—Jas. B. Baird, Atlanta.

42. Cicatricial Strictures of the Esophagus—Geo. R. White, Savannah.

ABSTRACT.—The difficulties met in the management of these cases are explained by a study of the pathology of the diseased organ. The high mortality is due to starvation and perforation of esophagus by bougies. Methods devised for the relief of this condition and the cause of failure of most of them. Inadvisability of attempting retrograde dilatation and esophagotomy. Kalder's gastrostomy recommended as a preliminary for nourishment and as an aid to treatment. A satisfactory method of dilation by the author's bougies making use of both the mouth and gastrostomy.

43. Hip Joint Operation, Formation of False Joint and Young Man Walking—J. T. Gammage, Pineview.

ABSTRACT.—Description of operation for tubercular hip, which produces such wonderful results in this case and previous cases.

44. Senile Changes in Bachelors—M. X. Corbin, Savannah.

45. Treatment of Fracture of the Clavicle—W. F. Westmoreland, Atlanta.

ABSTRACT.—The frequency of fracture of the clavicle. Difficulty of holding fractured ends in apposition, mechanical not muscular. Present methods usually unsatisfactory, and based upon erroneous conclusions. The essayist suggests plan based upon reduction by position and the application of permanent plaster of paris splints.

46. American Hookworm—A. G. Fort, Lumpkin.

ABSTRACT.—Deductions drawn from experience in treatment of 408 cases. Definition. History. Its prevalence in Southwest Georgia. Description of parasite. Description of eggs. Infective stage. Two modes of infection. The larvae. Suitable conditions for development. Ground itch. Race. Sex. Symptoms. Relative number of parasites and length of time of infection. Clinical picture. Diagnosis. Treatment. Preparation of patient. Diet. Thymalsetanophthal Number of treatments necessary. After treatment. Prophylaxis.

47. Practical Disinfection—C. J. Montgomery, Augusta.

ABSTRACT.—Meaning of common terms. Objects to be attained. Importance and limitation of physical agents. Chemical agents, liquid and gaseous; advantages and disadvantages of these commonly used. Hygienic management of infectious diseases. Disinfection of skin, feces, urine, sputum, etc. Treatment of crockery and silverware, bed-linen and clothing, books and furniture. Room disinfection.

48. Drainage in Suppurative Conditions About the Abdomen—W. S. Goldsmith, Atlanta.

49. The Personality of the Patient a Factor in Treatment—I. H. Adams, Macon.

ABSTRACT.—Medicine an art, as well as a science. Great advances in medicine have been along one line at a time. Therapeutics now in the ascendency. In treatment, we consider the causes of disease, the remedies used to combat disease, and the patient's reaction to the disease and the remedies used. Best results attend the efforts of the physician who treats the patient and not the disease. Train the powers of observation and in due time deductions will be drawn automatically. Face, form, and manner, give inestimable evidence of vital resistance.

AFTERNOON SESSION—THURSDAY.

2 to 6 O'clock.

50. Hereditary Multiple Osteomata—T. P. Waring and E. R. Corson, Savannah.

51. Some Remarks Concerning Deaf-Mutism—M. M. Stapler, Macon.

ABSTRACT.—Showing a probable cause of deaf-mutism due to closure of eustachian tubes, displacement of the stapes and exhaustion of the stapedius muscle and their correlation in producing the deafness. How to remove these causes and what results their removal has shown. Why we have not established hearing heretofore for mutes. Preconceived opinions and inherited prejudice as a handicap to the advance of original, scientific work for deaf-mutes.

52. Headaches and Neuralgia Due to Diseases of the Nose and Accessory Sinuses—Hugh M. Lokey, Atlanta.

ABSTRACT.—Headaches and neuralgia are not diseases per se—but are symptoms of morbid conditions, either at the seat of pain or referred through nerve reflexes from distant sources. The anatomical closeness of the nose and accessory sinuses, with poor drainage facilities of the maxillary antra and the ethmoidal and spheenoidal cells, with the closely anastomosed nerve supply, predispose to nerve irritation and headaches from diseases of these parts. While a large per cent. of these cases are operative, and positive diagnosis is sometimes impossible, there are many cases that can be treated and relieved by one who is not a specialist in rhinology. But the lesion should be sought out and treated, and the physician should not be content in treating the symptoms.

53. Spongy Angiomata, Congenital or Acquired—M. B. Hutchins, Atlanta.

ABSTRACT.—Angioma spongiosum a better name than angioma cavernosum, because cavernous parts often constitute less than half the growth. Old methods of treatment, as electrolysis, cautery, coagulants, or ligature often fail. Clean dissection, plastic repair best, where area is not too great. Acquired or traumatic, spongy, vascular lesions a new and rather different type, but of sufficient likeness to be included in the discussion. Treatment of these minor surgical.

54. Operation for Retrodeviation of the Uterus—E. C. Davis, Atlanta.

ABSTRACT.—First: The normal supports of the uterus. Second: Some of the causes operative to cause displacements. Third: Various operations which have been performed to relieve retro-deviation; first the formation of adhesions or the fixation of the uterus; second, ventro-suspension of the uterus or formation of an adventitious ligament; third, operations upon the broad ligaments; fourth, operations upon the utero-sacral ligament; fifth, operations upon the verico-uterine ligament; sixth, operations upon the round ligaments, and these sub-divided as follows: First, plication of the round ligaments; second, Alexander's operation and its various modifications; third, Mayo's operation; fourth, Dudley's operation; fifth, Noble's operation and its various modifications.

55. Pelvic Inflammation—Etiology, Prevention and Treatment—R. R. Kime, Atlanta.

56. Gastro-Jejunostomy—Report of Cases—Edward G. Jones, Atlanta.

ABSTRACT.—

(a) For pyloric stenosis.

(b) For chronic gastric ulcer without stenosis.

(c) For duodenal stenosis.

No loop employed in any case.

Absence of vomiting, relief from pain, etc., after these operations.

57. Treatment of Alcoholism and Drug Habit—A. L. R. Avant, Savannah.

ABSTRACT.—Lack of literature on this important subject. Paper based on daily observations. Plain drunk needs very little attention. Physicians usually call after constant drinking spells, and find the patient suffering from gastric disturbances. Treatment consists in cleansing stomach, saline purge, systematic treatment, liquid diet, and treatment of nervous system. Delirium tremens demands more energetic and persistent efforts. In drug habitues we endeavor to build up patient and improve moral stamina. Gradual withdrawal of drugs in severe cases, substitute other remedies if necessary. Secure pleasant surroundings for patient. Secure co-operation of patient.

58. The State's Attitude to the Drug Habitue—J. L. Frazer, Fitzgerald.

59. Treatment of Sprains—Theodore Toepel, Atlanta.

ABSTRACT.—Sprains. Kinds. Ligaments and tissues involved. Sprains in subjects of rheumatic or gouty habit; in strumous subjects. Treatment of ordinary sprains. Application of hot air. Importance of massage in treatment. Encouragement of activity between treatments. When the appliance of plaster bandage becomes necessary. Massage and plaster cast. Resistive exercises. Chronic sprains. Atrophy of muscle and immobility. Improved treatment.

60. The Best Point to Open the Abdomen in Operation on the Vermiform Appendix—L. C. Fisher, Atlanta.

61. Sarcoma of the Omentum, with Report of an Interesting Case—J. R. Garner, Atlanta.

62. Presentation of a New Eye Dressing—J. Lawton Hiers, Savannah.

FRIDAY MORNING, APRIL 17.

9 to 1 O'clock.

Report of House of Delegates.

READING OF PAPERS.

63. Milk: Its Relation to the Public—Ralph M. Thompson, Savannah.

64. Chloroform in Childbirth, and Some of the Accidents of Delivery—L. S. Osborne, Fitzgerald.

65. The Treatment of Crushes of the Extremities—Thos. H. Hancock, Atlanta.

ABSTRACT.—Shock caused from crushes; how to prevent it, and how to treat it. When to operate. How to amputate. How to feed these patients.

66. Anatomy of the Peritoneum and Gastro-Intestinal Tract from the Standpoint of Development—W. B. Armstrong, Atlanta.

67. A Successful Laminectomy, with Patient Present—Howard J. Williams, Macon.

68. Some remarks on Cholangitis—H. R. Donaldson, Atlanta.

69. Post-Operative Suppression of Urine—A. B. Cleborne, Savannah.

ABSTRACT.—The purpose of my paper is to show how little we know of (part 61, Suppression) pathologically and etiologically and to plead for more work to be done, in the dead-house and our experimental laboratories. Upon this we can only hope for a sound basis to write out our therapeutical ideas.

70. Indications and Technique of Infusion—Monroe Smith, Atlanta.

71. Suppuration of the Middle Ear—J. L. Jackson, Savannah.

72. Fractures of the Skull—W. A. Norton, Savannah.

ABSTRACT.—Description of extensive fractures of skull. Duret's principle. Contre-coup fracture of the base. Report of case of pressure on brain, causing interruption and suspension of thought. Report of case of great destruction of skull and brain tissue—complete recovery of mentality. Drainage from frontal sinus through nose. Summary.

73. Pyelitis in Pregnancy—F. G. Hodgson, Atlanta.

74. Our Duty to the Consumptive—L. C. Roughlin, Atlanta.

75. Meningitis—J. C. Luke, Ocilla.

76. Tumors of the Bladder—A. L. Fowler, Atlanta.
ABSTRACT.—Our knowledge of vesical tumors a recent acquisition. The cystoscope triumphant in the perfection of our diagnostic means. The grave danger when employed by the experienced. Some photographs.

77. Sacro-Iliac Disease—C. R. Andrews and Michael Hoke, Atlanta.
ABSTRACT.—The sacro-iliac synchondroses are true joints, capable of being affected as are other joints. Anatomic relations render these joints more liable to strain and displacement. Importance to the general practitioner, obstetrician and gyecologist. Toxic inflammation superimposed on chronic and acute conditions of the sacro-iliac joints.

78. Typhoid fever—E. J. Dorminy, Fitzgerald.

79. Two interesting cases of Mastoiditis—J. H. Crawford, Atlanta.
ABSTRACT.—First. Primary mastoiditis of traumatic origin. Rare occurrence of primary mastoiditis in comparison to secondary. Percentage of cases originating as primary condition. Report of case. Sight of injury. Symptoms, etc. Description of operation. Remarks on anatomical structure and relation of mastoid cells to the middle ear favoring extension of infection. statistical summaries.
Second. Case of mastoiditis occurring in babe eleven months old. Structure of process in infants. Report of case. Unusual development of mastoid cells. Operation.

80. Post-Graduate Study in Europe, with Reference to Eye, Ear, Nose and Throat—O. H. Johnson, Athens.

ABSTRACT.—General consideration. Advantages of European study. Where to go. Comparison of opportunities in different European medical centers. How long to stay. Living expenses. Cost of courses and tuition. Traveling expenses. Suggestions as to different routes by land and sea, and hints to the traveler.

81. Significance of Arterial Hypertension—Its Treatment —Ralston Lattimore, Savannah.

ABSTRACT.—

1. Arterial hypertension in arteriosclerosis.
2. Arterial hypertension in nephritis.
3. Arterial hypertension without nephritis.
4. Arterial hypertension of unknown origin.
5. Arterial hypertension as a compensatory manifestation.
6. Effects and danger of arterial hypertension.
7. Therapeutic indications.
   (a) preventive treatment.
   (b) Adjuvant treatment.
   (c) Emergent treatment.

AFTERNOON SESSION.
3 O'clock.

Election of officers was as follows: President, Thomas D. Coleman, of Augusta; First Vice-President, W. B. Armstrong, Atlanta; Second Vice-President, Ralston Lattimore, Savannah; Secretary-Treasurer, Claude A. Smith, Atlanta; Delegates to the American Medical Association, Drs. H. F. Harris, Atlanta, and W. W. Owen, Savannah. The next meeting will be held in Macon.

---

### NAMES AND ADDRESSES OF THE SECRETARIES OF THE VARIOUS STATE EXAMINING BOARDS.

---

Arizona, Dr. W. H. Sanders, Montgomery.

Alabama, Dr. Ancil Martin, Phœnix.

Arkansas (R), Dr. F. T. Murphy, Brinkley.

(H) Dr. V. H. Hallman, Hot Springs.

(E) Dr. A. J. Widener, Little Rock.

California, Dr. Charles L. Tisdale, Alameda.

Colorado, Dr. S. D. Van Meter, 1723 Tremont St., Denver.
Connecticut (R), Dr. Chas. A. Tuttle, New Haven.
(H) Dr. E. C. M. Hall, 82 Grand Avenue, New Haven.
(E) Dr. T. S. Hodge, 16 Main Street, Torrington.
Delaware, Dr. P. W. Tomlinson, Wilmington.
District of Columbia, Dr. George C. Ober, Washington.
Florida (R), Dr. J. D. Fernandez, Jacksonville.
(H) Dr. C. W. Johnson, Jacksonville.
Georgia (R), Dr. E. R. Anthony, Griffin.
(H) Dr. R. E. Hinman, Atlanta.
(E) Dr. C. H. Field, Marietta.
Hawaii, Dr. George Herbert, Ilakea St., Honolulu.
Idaho, Dr. W. F. Howard, Pocatello.
Illinois, Dr. J. A. Egan, Springfield.
Indiana, Dr. W. T. Gott, 120 State House, Indianapolis.
Indian Territory.
Central District, Dr. J. B. Smith, Durant.
Northern District, Dr. D. F. Fortner, Vinita.
Southern District, Dr. W. L. Peters, Chickasha.
Western District, Dr. M. L. Williams, Muskogee.
Iowa, Dr. Louis A. Thomas, State House, Des Moines.
Kansas, Dr. D. P. Cook, Clay Center.
Kentucky, Dr. J. N. McCormack, Bowling Green.
Louisiana (R), Dr. F. A. LaRue, 211 Camp Street, New Orleans.
(H) Dr. Gayle Aiken, New Orleans.
Maine, Dr. Wm. J. Maybury, Saco.
Maryland, (R), Dr. J. P. McP. Scott, Hagerstown.
(H) Dr. Jos. S. Garrison, 848 W. North Avenue, Baltimore, Md.
Massachusetts, Dr. E. B. Harvey, State House, Boston.
Michigan, Dr. B. D. Harrison, 205 Whitney Building, Detroit.
Minnesota, Dr. W. H. Fullerton, St. Paul.
Mississippi, Dr. J. F. Hunter, Jackson.
Missouri, Dr. J. A. B. Adcock, Warrensburg.
Montana, Dr. Wm. C. Riddell, Helena.
Nebraska, Dr. E. J. C. Sward, Oakland.
Nevada, Dr. S. L. Lee, Carson City.
New Hampshire (Regent) H. C. Morrison, Concord.

(R) Dr. J. T. Greeley, Nashua.
(H) Dr. R. V. Sweet, Rochester.
New Jersey, Dr. J. W. Bennett, Long Branch.
New Mexico, Dr. J. A. Massie, Santa Fe.
New York, (Laws), Dr. Howard J. Rogers, Albany.
(Examinations) Dr. C. F. Wheelock, Albany.
Dr. Maurice J. Lewi, 501 Fayette Park, New York City.
North Carolina, Dr. G. T. Sikes, Grissom.
North Dakota, Dr. H. M. Wheeler, Grand Forks.
Ohio, Dr. George H. Matson, Columbus.
Oklahoma, Dr. J. W. Baker, Enid.
Oregon, Dr. B. E. Miller, The Dekum, Portland.
Pennsylvania (Council), Dr. N. C. Shæffer, Harrisburg.
(R) Dr. Winters D. Hamacker, Meadville.
(H) Dr. Joseph C. Guernsey, Philadelphia.
(E) Dr. W. H. Blake, Philadelphia.
Philippine Islands, Dr. F. W. Dudley, Manili.
Porto Rico:
(Bd. of Health), Dr. Wm. F. Smith, San Juan.
(Bd. of Exam's.), Dr. Quevedo Baez, San Juan.
Rhode Island, Dr. Gardner T. Swarts, Providence.
South Carolina (R), Dr. W. M. Lester, Columbia.
South Dakota, Dr. H. E. McNutt, Aberdeen.
Tennessee, Dr. T. J. Happel, Trenton.
Texas, Dr. G. B. Foscue, Waco.
Utah, Dr. R. W. Fisher, Salt Lake City.
Vermont, Dr. W. Scott Nay, Underhill.
Virginia, Dr. R. S. Martin, Stuart.
Washington, Dr. C. W. Sharples, Seattle.
West Virginia, Dr. H. A. Barbee, Point Pleasant.
Wisconsin, Dr. J. V. Stevens, Jefferson.
Wyoming, Dr. S. B. Miller, Laramie.

—*St. Louis Medical Review, March,* 1908.

---

### FIFTH PAN-AMERICAN MEDICAL CONGRESS.

---

To the Medical Profession.

Gentlemen:

The government and the people of the Republic of Guatemala, as well as the National Committee of the Fifth Pan-American Medical Congress are actively endeavoring to do all in their power, in a sure and efficient way, to make this meeting a great success.

With this object in view the Committee will be pleased to invite you personally to attend, as well as the members of the Society or Medical Fraternity to which you belong, in order that through your presence and works the certain success of the Congress, that science expects of its representatives, will be assured.

The Committee hopes that you and the other members of your institutions will meet at Guatemala on the 5th, 6th, 7th, 8th, 9th and 10th of August, 1908, and sincerely begs that from now on you will not hesitate to keep yourself in fraternal relations with this Committee, and also that you will let us know beforehand if you intend to attend the Congress in person or to send some scientific contributions.

The Committee hopes to receive a reply shortly to the invitation to this meeting, which should serve as an incentive to unite professional interests, to stimulate the advance of medical science and to contribute to the preservation of the health and the prolongation of the life of the people of the Americas.

We take advantage of this opportunity to express to you the best regards of,

Yours very truly,
Juan J. Orteaga, Pres.
Jose Azurdia, Sec'y.

---

### NOTICE TO ALUMNI OF THE TULANE MEDICAL DEPARTMENT

---

It is important that all graduates of Tulane intending to be present at the meeting of the A. M. A. in Chicago, June 2nd to 5th, should write at once to Dr. Hugh B. Williams, No. 100 State street, for information concerning the gathering of the Alumni on June 2nd. Tulane headquarters will be at the Auditorium Hotel and Alumni are urged to call, upon their arrival, for information. This is important.

The American Medical Editor's Association will convene in its thirty-ninth annual session in Chicago, May 30th and June 1st, in the Auditorium hotel.

---

The annual meeting of the Medical Society of the State of California was held at Coronado, April 21st to 23rd, inclusive.

---

The Thirty-fourth Annual Report of the Cincinnati Sanitorium, for the year ending November 30th, 1907, is received from Dr. Frank W. Langdon, the Medical Director. The year has been a successful one with 208 patients, 123 males and 85 females, 20 of whom were from Indiana, making 413 from our State since the beginning.

---

The Charlotte Medical Journal and the Carolina Medical Journal have been consolidated, and will be published hereafter as the *Charlotte Medical Journal.* The editorial and business management will remain under the direction of Dr. E. C. Register, who has heretofore conducted the Charlotte Medical Journal.

---

"Bone cases" should not be dressed too often after operation. The fine granulations which form are very liable to be pulled off with the removal of the packing.—*American Journal of Surgery.*

---

### REGULAR MEETING, FULTON COUNTY MEDICAL SOCIETY, APRIL 2, 1908.

EDITED BY R. R. DALY, M. D.

Dr. Hutchins presented a case of extra shrdlueta and innocent syphilitic infection. The chancre was in the face. It gave history of cutting when shaving. Diagnosis had been confirmed as far as possible by microscopic examination, though, as Dr. Ballenger said, the sperochetae pallida was hard to find because of the difficulty in getting a proper smear until sealed dressing gave sufficient retained secretion.

Dr. P. Calhoun gave an interesting and comprehensive paper upon "A Synopsis of Personal Observation in 271 Mastoid Operations." (Published in full in our last issue.)

Dr. Toepel read a paper on "Sprains," which was well received.

Discussed by Dr. Hoke, Dr. Goldsmith, Dr. Hancock and Dr. Ogglesby.

---

### FULTON COUNTY MEDICAL SOCIETY, MARCH 19.

---

Dr. Armstrong exhibited a case of bilateral malignant growth of breasts of female.

Dr. Hutchins said he had never seen a case so extensive as this one. There was some history of cancer in the family, but it was also possible that the etiology lay in specific gummata.

Dr. Hutchins re-exhibited his case of extra genital syphilitic infection. The orginal lesion is breaking down somewhat. Macu lar eruption has appeared and there is a fine, milliary, papular condition added. There is no gland involvement as yet.

Dr. J. E. Paulin read an excellent paper upon "The Opsonic Contents of the Body Fluids." Published in our last issue.

Discussed by Dr. McCrea, Dr. Andrews, Dr. Hutchins and Dr. Armstrong.

Dr. W. B. Armstrong gave an illustrating "Demonstration of the Anatomy of the Peritoneum and the Gastro Intestinal Tract," having special relation to the anamolies of position of the appendix. It was favorably discussed by Dr. McCrea, Dr. J. L. Campbell, Dr. Strickler, Dr. Kime and Dr. Clark.

